# Cytomegalovirus reactivation and its clinical impact in patients with solid tumors

**DOI:** 10.1186/s13027-015-0039-4

**Published:** 2015-12-03

**Authors:** Konstantin Schlick, Michael Grundbichler, Jutta Auberger, Jan Marco Kern, Markus Hell, Florian Hohla, Georg Hopfinger, Richard Greil

**Affiliations:** Department of Internal Medicine III with Hematology, Medical Oncology, Hemostaseology, Infectious Diseases, Rheumatology, Oncologic Center, Laboratory of Immunological Molecular Cancer Research, Paracelsus Private Medical University Salzburg, Salzburg, Austria; Division of Medical Microbiology of the Department of Laboratory Medicine, Paracelsus Medical University Salzburg, Salzburg, Austria; Department of Hospital Epidemiology and Infection Control, Paracelsus Private Medical University Salzburg, Salzburg, Austria; Department Onkology, Allgemeines Krankenhaus-Universitätskliniken, Wien, Austria

**Keywords:** Cytomegalovirus infection, CMV, EBV, Solid tumor

## Abstract

Cytomegalovirus reactivation can be life threatening. However, little evidence on its incidence in solid cancers is available. Therefore our single center Cytomegalovirus polymerase chain reaction database with altogether 890 CMV positive blood serum samples of mainly hematological and oncological patients was retrospectively analyzed to examine the occurrence of Cytomegalovirus reactivation in patients with solid tumors, resulting in 107 patients tested positive for Cytomegalovirus reactivation. Seventeen patients with solid cancer and a positive CMV-PCR test were identified, of which eight patients had clinically relevant CMV disease and received prompt antiviral treatment. Five patients fully recovered, but despite prompt antiviral treatment three patients died. Among these three patients two had significant co-infections (in one case EBV and in the other case Aspergillus) indicating that that CMV reactivation was at least one factor contributing to sepsis. The patient with the EBV co-infection was treated in an adjuvant therapy setting for breast cancer and died due to Cytomegalovirus and Epstein-Barr virus associated pneumonia despite intensive therapy. The other two patients had progressive disease of an underlying pancreatic cancer at the time of CMV diagnosis. One patient died due to attendant uncontrollable Aspergillus pneumonia, the other patient most likely died independent from CMV disease because of massively progressive underlying disease.

Cytomegalovirus reactivation and disease might be underestimated in routine clinical practice. In our retrospective analysis we show that approximately 50 % of our patients suffering from solid cancers with a positive Cytomegalovirus polymerase chain reaction also had clinically relevant Cytomegalovirus disease requiring antiviral therapy.

## Background

Cytomegalovirus (CMV) reactivation especially in immunocompromised patients may rapidly progress to a fatal CMV disease associated with significant morbidity and mortality [1–3]. However only limited data is available on the role of CMV reactivation/disease in patients with solid cancers e.g. under chemotherapeutic treatment. In our clinical experience CMV reactivation is an important differential diagnosis in the infectiological work up of these patients. Guidlines on this subject are not jet available To our knowledge nowadays only a few small case reports or post-mortem analyses are available: A single center study that analyzed 107 patients with CMV disease, including 75 with solid cancer, reported a mortality rate of 61.3 % [4]. In a retrospective post-mortem analysis of 47 cancer patients, with a biopsy-positive gastro intestinal CMV disease,13 had an underlying solid cancer [5]. Further death attributable to CMV was reported in 42 % of a study cohort that included hematological and solid tumor manifestations [6]. These data suggest that there exists a reliable risk of CMV reactivation/disease in solid cancer patients. We investigated the incidence and impact of CMV reactivation in solid cancer patients by performing a retrospective analysis of our single center CMV database.

The database was generated by evaluation of all CMV-PCR analyses done during the infectiological work up of our mainly hematological and oncological patients treated between January 2007 and October 2012. Altogether 890 blood serum samples were collected and analyzed by qualitative and quantitative PCR. Positivity in the qualitative CMV-specific real time PCR assay (Artus CMV PCR kit, Qiagen, Hilden, Germany) of serum was defined as CMV viremia with a cut off of > 20 copies/ml. In case of CMV positivity indicating clinical relevant CMV infection a tissue specimen was as far as possible depending on the clinical features gained by bronchoscopy (pneumonitis, e.g.) or endoscopy (gastrointestinal symptoms, e.g. diarrhea).

### Retrospective database analysis

CMV – PCR positivity as defined was detected in 107 of our hematological and oncological patients. Of these 107 patients 17 patients had a solid cancer; the remaining 90 patients had underlying hematological diseases (e.g. Acute Myeloid Leukemia, Chronic Lymphatic Leukemia and Non-Hodgkin Lymphoma) and/or HIV infection. In our further analysis we concentrated on the patients with solid cancers.

The median age of the patients in the solid cancer cohort with a positive CMV PCR was 70.6 years (range, 52–93 years). The eight patients who developed clinically relevant CMV disease were slightly older, with a median age of 71,6 years (range, 63–85 years) and had a higher median copy number of 6813 copies/ml (range, 476–18,000 copies/ml) compared to 4590 copies/ml (range, 34–18,000 copies/ml) in the whole solid cancer CMV PCR positive cohort.

At the time of diagnosis 15 of the 17 patients with solid cancers underwent chemotherapy. Five patients received chemotherapy in an adjuvant setting. Ten patients were treated within a palliative setting for metastasized disease (Most of these patients already underwent multiple courses of chemotherapy before). One patient was solely treated with anti-hormonal therapy in a palliative setting, never receiving any kind of chemotherapy before. One additional patient in an adjuvant setting did not receive any kind of chemotherapy at the time of reactivation. Primary tumor sites and their distribution were as follows: breast cancer in six patients, pancreatic cancer in three, carcinoma of unknown origin in three, squamous cell lung cancer in two, small cell lung cancer in two and esophageal cancer in one patient.

Co-Infections at the time of CMV-positivity were given in 9 of 17 patients with Aspergillus in two patients, Candida in one patient, Pseudomonas aeruginosa in one patient, Epstein-Barr virus (EBV) in four patients and Herpes-Simplex virus (HSV) in two patients. During the detection of CMV positivity, four patients had an absolute neutrophil count of < 500/μl, nine patients showed elevated C-reactive protein levels (>10 mg/dl) at the time of CMV PCR positivity detection. Because most patients were in an advanced disease stage, several comorbidities and a history of infectious complications during the course of chemotherapy were observed, as well as hypoalbuminemia. Comorbidities in the patient cohort were distributed as follows: the most common comorbidity was hypertension in four patients, followed by chronic obstructive pulmonary disease in two patients, cerebral insult and diabetes mellitus II in one patient, and nutritive toxic liver cirrhosis in one patient. Quantitative data on immunoglobulin (Ig) G status were available in four patients, one patient showed a decrease to < 400 mg/dl (normal range: 700–1600 mg/dl).

The median time from cancer diagnosis to overt CMV disease was 5.6 months (range, 1–24 months) and time to recovery or death from CMV infection ranged between 3 and 5 weeks. Table 1 shows the characteristics of these 17 patients in more detail.

**Table 1:** Patients characteristics

Gender	Age	Tumor entity	TX setting	Tumor therapy	CMV copies/mL	Assay (specimen)	Kind of co-infection	CMV therapy	Outcome
f	59	Breast	adjuvant	no	235	blood	no	no	alive
m	59	SCLC	palliative	CTX	132	blood	no	no	alive
f	79	SCLC	palliative	CTX	34	blood	HSV-esophagitis	no	alive
f	93	Breast	palliative	anti-hormonal	120	blood	no	no	alive
m	70	SCC	adjuvant	CTX/RTX	62	blood	no	no	alive
m	52	SCC	palliative	CTX/RTX	7,650	blood	no	no	alive
f	53	Breast	palliative	CTX	8,000	blood	no	no	alive
m	76	CUP	palliative	CTX	129	blood	no	no	alive
f	63	Pancreas	palliative	CTX	34	blood	no	no	alive
m	65	Pancreas	palliative	CTX	3,409	blood	EBV, candida, pseudomonas	no	death
m	85	Esophagus	palliative	CTX	4,500	blood	no	gancyclovir	alive
f	65	Breast/Lung	adjuvant	CTX	14,000	blood & BAL	EBV, HSV	gancyclovir	alive
m	67	CUP	palliative	CTX	18,000	blood	no	gancyclovir	alive
f	72	CUP	palliative	CTX	8434	blood	no	gancyclovir	alive
f	63	Breast	adjuvant	CTX	476	blood	no	gancyclovir	alive
f	81	Pancreas	palliative	CTX	6,000	blood	Aspergillus, EBV, candida	gancyclovir	death
f	69	Breast	adjuvant	CTX	6,810	blood & BAL	Aspergillus, EBV	gancyclovir	death

*BAL* bronchioloalveolar lavage, *CMV* cytomegalovirus, *CTX* chemotherapy, *CUP* carcinoma of unknown origin, *EBV* Epstein-Barr virus, *HSV* herpes-simplex virus, *RTX* radiotherapy, *SCC* squamous cell cancer, *SCLC* small cell lung cancer

Clinically apparent CMV disease was diagnosed in 8 of 17 patients with CMV-PCR positivity. CMV disease was considered to be clinically relevant when fever of unknown origin persisted despite broad antibiotic +/− antimycotic treatment and CMV-PCR positivity was given. In these cases, antiviral therapy was promptly started with intravenous ganciclovir 5 mg/m^2^ twice a day. After start of antiviral therapy longitudinal CMV-PCR quantifications under antiviral treatment were done. In 5 of 8 patients the CMV copy numbers reduced to within normal range under antiviral therapy. All these five patients survived their infectious complication. In the remaining three patients the longitudinal CMV copy numbers after start of therapy could not be obtained due to fulminante clinical course. Of these three patients one patient died because of infectious complications of CMV and attendant EBV reactivation, one died due to CMV and Aspergillus infection, and one patient died from massively progressive underlying disease most likely independent from CMV disease before getting any specific antiviral therapy. A more detailed description of the three patients who succumbed to CMV disease is included below in the case presentation section.

## Case presentation

### Case 1

The patient was a 65-year-old man with newly diagnosed adenocarcinoma of the pancreatic tract with hepatic, peritoneal and pulmonary metastases. After a single course of palliative chemotherapy with gemcitabine (1000 mg/m^2^) the patient was hospitalized with high fever and a poor general health status. Within 2 h after hospitalization, he developed sepsis with organ failure before starting any specific therapy. Post-mortem blood cultures were only positive for CMV with a copy number of 3409 copies/ml detected by CMV-PCR. No further pathogen was detected in the blood cultures.

### Case 2

The patient was a 70-year-old woman who had been receiving adjuvant therapy for triple negative breast cancer diagnosed in August 2010. She received three cycles of epirubicin (60 mg/m^2^) and cyclophosphamide (60 mg/m^2^), as well as two applications of taxotere (75 mg/m^2^) with G-CSF prophylaxis. After the second cycle of paclitaxel, she experienced fever of 38.8 °C. An intensive clinical work-up including laboratory testing, chest x-ray, and blood cultures was performed without gaining any infectiological focus or pathogen except positivity for CMV and EBV-PCR. Treatment with ganciclovir and specific immunoglobulin was promptly started. However, the patient’s respiratory status deteriorated and she had to be transferred to the intensive care unit (ICU), where the CMV and EBV pneumonia was confirmed by bronchoscopy. The patient died from GI-bleeding 16 days after the CMV and EBV infection became evident.

### Case 3

The patient was a 60-year-old woman with metastatic pancreatic carcinoma who was under palliative therapy with 5-FU, leucovorin, irinotecan and oxaliplatin. After a single course of chemotherapy, the patient developed multiple pulmonary infections resulting in cardiorespiratory insufficiency leading to an ICU admission. The microbiological work-up resulted in the detection of Aspergillus, herpes simplex virus (HSV), EBV and CMV in BAL specimens. Despite intensive antimicrobial therapy including ganciclovir, the patient succumbed to uncontrollable Aspergillus pneumonia.

## Conclusion

CMV disease is a well-known complication in immunocompromised or organ transplanted patients. However, little evidence is available regarding the incidence of CMV disease in patients with solid cancers. In the present single center retrospective analysis, we showed the clinical impact of CMV reactivation and viremia in solid tumor patients. Accumulating data suggests that CMV disease in these patients is more frequent than previously estimated. Furthermore it has to be pointed out that CMV testing is not routinely done in clinical practice and that therefore CMV reactivation or disease may be underreported. Viral load to be of significance was considered to be above >1000copies/ml, due to a lack of guidlines. In the current manuscript the average viral load is > 4000copies /ml.

Our data show that approximately 50 % of patients with CMV PCR positivity developed clinically relevant CMV-viremia, requiring specific therapy. The early administration of specific antiviral treatment may improve the outcome of these patients and may avoid unsuccessful antibiotic therapy and prolonged hospitalization. Clinicians should be aware of the broad range of potential complications of CMV infection in these patients.

We therefore propose the inclusion of routine CMV screening in solid cancer patients presenting with fever or unknown origin. Larger studies are necessary to identify the risk factors of developing CMV disease. The increase in the number of elderly patients receiving chemotherapy and the fact that CMV prevalence increases with age suggests that CMV reaction and CMV disease are treatment complications that will increase in frequency in the future.

## Consent

The current retrospective analysis underwent critical evaluation by the local ethical committee regarding the necessity of an informed written consent. Accordingly, it was concluded that written IC was not needed due to the retrospective and non-invasive character of the current analysis. Moreover, none of the patients was alive at time of evaluation.

## Abbreviations

CMV: Cytomegalo virus
EPV: Epstein Barr virus
PCR: Polymerase chain reaction
HSV: Herpes-Simplex- Virus
BAL: Bronchioloalveolar lavage I
GI: Gastro intestinal
